# P05.61. The multidimensional assessment of interoceptive awareness (MAIA)

**DOI:** 10.1186/1472-6882-12-S1-P421

**Published:** 2012-06-12

**Authors:** WE Mehling, C Price, J Daubenmier, E Bartmess, M Acree, V Gopisetty, A Stewart

**Affiliations:** 1 University of California, San Francisco, San Francisco, USA; 2 University of Washington, Seattle, USA

## Purpose

Body awareness and interoceptive awareness are closely related constructs proposed to mediate the health benefits of mind-body therapies, e.g. mindfulness meditation, yoga, Tai Chi, Feldenkrais and others. By integrating psychological and neuroscientific concepts with experiential phenomenology and by distinguishing multiple dimensions, contradictory construct interpretations are clarified. However, no self-report instruments are available that can be used to distinguish between beneficial and maladaptive forms of body awareness. We describe the development of the Multidimensional Assessment of Interoceptive Awareness (MAIA) scales.

## Methods

The systematic process involved reviewing the current literature, specifying a multi-dimensional conceptual framework, evaluating prior instruments, developing items, and conducting focus groups with instructors and patients of body awareness-enhancing therapies. Following refinement by cognitive pre-testing, items were field-tested in 332 students and instructors of mind-body approaches: 123 meditation, 107 yoga/tai chi, 47 somatic therapies (Feldenkrais, Alexander, Breath, body-oriented psychotherapy), and 55 massage. Final item selection was achieved by analyzing field-test data in an iterative process using multiple methods, including exploratory cluster and confirmatory factor analyses, comparison between known groups, and correlations with established measures of related constructs.

## Results

The resulting 32-item multi-dimensional instrument assesses eight concepts: Noticing, Distracting, Worrying, Attention Regulation, Emotional Awareness, Self-Regulation, Body Listening and Trusting. Internal consistency of the 3-7-item sub-scales ranges from .66 to .87, inter-scale correlations from .09 to .60. The confirmatory factor analysis showed acceptable model fit (RMSEA .060; CFI .886) with good item-scale correlations (all p<0.001) and negligible modification indices. Hypotheses for correlations with measures of related constructs were generally confirmed. The measure was able to distinguish between more and less experienced practitioners of mind-body approaches and between different therapeutic modalities, which vary in their focus on aspects of interoceptive awareness.

## Conclusion

The psychometric properties of these final scales suggest that the MAIA may serve as starting point for research and further collaborative refinement.

